# Dynamical Analysis of a New Chaotic Fractional Discrete-Time System and Its Control

**DOI:** 10.3390/e22121344

**Published:** 2020-11-27

**Authors:** A. Othman Almatroud, Amina-Aicha Khennaoui, Adel Ouannas, Giuseppe Grassi, M. Mossa Al-sawalha, Ahlem Gasri

**Affiliations:** 1 Department of Mathematics, Faculty of Science, University of Ha’il, Ha’il 81451, Saudi Arabia; o.Almatroud@uoh.edu.sa (A.O.A.); m.alswalha@uoh.edu.sa (M.M.A.-s.); 2 Laboratory of Dynamical Systems and Control, University of Larbi Ben M’hidi, Oum El Bouaghi 04000, Algeria; 3 Department of Mathematics and Computer Science, University of Larbi Ben M’hidi, Oum El Bouaghi 04000, Algeria; Ouannas.adel@univ-oeb.dz; 4 Dipartimento Ingegneria Innovazione, Universita del Salento, 73100 Lecce, Italy; giuseppe.grassi@unisalento.it; 5 Department of Mathematics, University of Larbi Tebessi, Tebessa 12002, Algeria; ahlem.gasri@univ-tebessa.dz

**Keywords:** chaos, discrete fractional calculus, coexisting attractors, 0–1 test, entropy, control

## Abstract

This article proposes a new fractional-order discrete-time chaotic system, without equilibria, included two quadratic nonlinearities terms. The dynamics of this system were experimentally investigated via bifurcation diagrams and largest Lyapunov exponent. Besides, some chaotic tests such as the 0–1 test and approximate entropy (ApEn) were included to detect the performance of our numerical results. Furthermore, a valid control method of stabilization is introduced to regulate the proposed system in such a way as to force all its states to adaptively tend toward the equilibrium point at zero. All theoretical findings in this work have been verified numerically using MATLAB software package.

## 1. Introduction

After the disclosure of Lorenz system in one of Lorenz’s papers on climate expectation [1], numerous researchers and analysts have broadly studied the chaotic dynamical system due to its various applications in engineering and applied science. Over a long time, several researchers proposed multiple discrete-time systems, such as the so-called logistic [2], Hénon’s [3], Arnold’s-cat, Lozi’s and tent maps [4]. In such maps, all chaotic attractors belong to the foremost common sort of attractors, for which the initial conditions and the unstable fixed points are both located near together [5]. These attractors are called self-excited due to being promptly found [5]. However, chaos can be generated also in unusual systems, for example, in systems with no equilibria or with fixed equilibria [6], for which the initial condition can only be found via extensive numerical searching. The attractors generated by these systems are called hidden attractors. The topic of chaotic maps characterized by “hidden attractors” has been only recently investigated [6,7,8,9,10]. For example, in [7] Jafri et al. studied a new 1D chaotic discontinuous map without inspiration from a logistic map, whereas in [6,8] 2D and 3D chaotic maps with different types of stable equilibria have been proposed. Moreover, in [9] 2D chaotic quadratic maps without equilibria and with no discontinuity in the right-hand equations have been introduced. Some other examples of chaotic attractors with curve equilibrium were numerically presented in [10]. These studies have proven the significant role of hidden attractors in theoretical problems and engineering applications. For example, hidden attractor can generate unexpected and potentially disastrous responses to perturbations in structures such as bridges and airplane wings. This is one of the main motivations that still inspires many researchers to devote their efforts in order to design new two or three-dimensional discrete-time chaotic systems with hidden attractors.

In recent years, attention has been focused on fractional calculus and fractional difference operators [11,12,13]. Referring to discrete fractional calculus, several types of difference fractional operators have been introduced over the years [14,15]. Recently, modeling numerous chaotic phenomena in the form of fractional-order systems, described by difference equations, has drawn the attention of many researchers. Researchers have extensively examined the potential applications of these maps in many fields, such as engineering, economics and others [16,17]. For this purpose, many fractional maps have been reported in the literature to show the different dynamical phenomena, for example, the fractional 3D generalized Hénon map that can be found in reference [18] and the Stefanski, Rossler and Wang fractional maps in [19]; the fractional discrete double scroll can be found in [20], and the dynamics of the fractional Grassi-Miller map are in reference [21]. Note that all these fractional-order maps show only attractors belonging to the self-excited ones. From what has been reported in the literature, the dynamical behavior of fractional-order discrete-time systems is heavily dependent on the fractional order, which introduces a new degree of freedom and makes them more suitable for secure communication and encryption, which has inspired researchers to devote themselves to the design of new two and three-dimensional fractional discrete-time chaotic systems with hidden attractors. To the best of our knowledge, few studies have been devoted in handling the fractional-order discrete-time (FoDT) systems without fixed points. The only three-dimensional chaotic system of fractional-order without equilibria has been proposed and reported in [22]. Said proposed system has motivated us to study and discuss a new three-dimensional FoDT system with no equilibrium points. Based on this consideration, this paper makes a contribution to the topic of fractional-order discrete-time systems with “hidden attractors” by presenting a new three dimensional discrete time system with no equilibria. The conceived system possesses an interesting property not explored in literature so far; i.e., it is characterized, for various fractional-order values, by the coexistence of various kinds of periodic and chaotic attractors. Moreover, the effects of the fractional-order, say *r*, on the type and the range of chaotic behavior of the FoDT system, are examined together with its dynamics. Besides, some tests of chaos, such as the 0–1 test and approximate entropy (ApEn), are included to show the performance of our numerical results. Furthermore, a control scheme is introduced to stabilize the trajectories of the system under consideration.

## 2. Necessary Discrete Fractional Operators

In this section, some preliminaries and basic concepts associated with discrete fractional calculus are presented. In the following definitions, we consider the time scale, $N_{a}=\left\{a,\;a+1,\;a+2,\;\ldots \right\}$, where a∈R.

**Definition** **1.** *[23,24] Let $X\left(t\right):N_{a}\to R$. The $r^{th}$-fractional-order sum is defined by:*

(1) $$\Delta_{a}^{-r}X\left(t\right)=\frac{1}{\Gamma \left(r\right)}\sum_{s=a}^{t-r}{\left(t-s-1\right)}^{\left(r-1\right)}X\left(s\right),$$

*where r>0 and $t\in N_{a+r}$. The term $t^{\left(r\right)}$ is the falling function which may be defined via gamma function, *Γ*, as follows:*

(2) $$t^{\left(r\right)}=\frac{\Gamma \left(t+1\right)}{\Gamma \left(t+1-r\right)}.$$

**Definition** **2.** *[13,15,25,26,27] Let X denote any function defined in $N_{a+n-r}$. The Caputo difference operator with order r∉N is defined by:*

(3) $${}^{C}\Delta_{a}^{r}X\left(t\right)=\Delta_{a}^{-(n-r)}\Delta^{r}X\left(t\right)=\frac{1}{\Gamma \left(n-r\right)}\sum_{s=a}^{t-\left(n-r\right)}{\left(t-s-1\right)}^{\left(n-r-1\right)}\Delta_{s}^{n}X\left(s\right),$$

*for $n=\left\lceil r\right\rceil +1$.*

## 3. The New FoDT System

In [8] the authors Haibo Jiang et al. preformed a schematic approach to find three-dimensional integer-order chaotic maps with no equilibrium or with stable equilibria. In this approach, an exhaustive computer search was carried out to obtain the “elegant” chaotic cases by considering numerous combinations of the coefficients and initial conditions [28]. Motivated by this strategy, we consider the following integer-order system:

(4) $$\left\{\begin{array}{c} x(n+1)=y(n),\\ y(n+1)=z(n),\\ z(n+1)=\alpha x(n)+\beta z(n)-x(n)y(n)+x(n)z(n)+\gamma . \end{array}\right.$$

Inspired by system (4), we remove and added some terms to obtain the following fractional discrete-time system [29]:

(5) $$\left\{\begin{array}{c} {}^{C}\Delta_{a}^{r}x\left(t\right)=-x\left(t-1+r\right)+y\left(t-1+r\right),\\ {}^{C}\Delta_{a}^{r}y\left(t\right)=-y\left(t-1+r\right)+z\left(t-1+r\right),\\ {}^{C}\Delta_{a}^{r}z\left(t\right)=\alpha x\left(t-1+r\right)+\beta z\left(t-1+r\right)-x\left(t-1+r\right)y\left(t-1+r\right)\\ \;\;\;\;\;\;\;\;\;\;\;\;\;\;\;\;\;\;\;\;\;\;\;\;\;\;\;\;\;\;\;\;\;\;\;\;\;\;\;\;\;\;\;\;\;\;\;\;\;+x(t-1+r)z(t-1+r)+\gamma , \end{array}\right.$$

where x,y and *z* indicate all states of system (5) and α,β and γ are the bifurcation parameters. For calculating the equilibrium points of this system, the following condition should be satisfied:

(6) $$\left\{\begin{array}{c} -x_{e}+y_{e}=0,\\ -y_{e}+z_{e}=0,\\ \alpha x_{e}+\beta z_{e}-x_{e}y_{e}+x_{e}z_{e}+\gamma =0, \end{array}\right.$$

where γ≠0. Here, we need to determine the case in which there are no real solutions for the following equation:

(7) $$\left(\alpha +\beta \right)x_{e}+\gamma =0.$$

Note that when α+β=0, then (7) will have no real solutions; therefore, there are no equilibrium points for system (5). Moreover, imposing the condition α+β=0 on the parameters of said system yields the hidden attractors. Before exhibiting the major results of this work, one significant theorem (Theorem 1) should be stated. This theorem is considered as a cornerstone for the next numerical method, and it will be, surely, required when we deal with system (5). For a full overview about this theorem and its proof, refer to [30].

**Theorem** **1.** *Consider the following fractional-order difference equations:*

(8) $$\left\{\begin{array}{c} {}^{C}\Delta_{a}^{r}u\left(t\right)=f\left(t+r-1,u\left(t+r-1\right)\right),\\ \Delta^{k}u\left(a\right)=u_{k},\;n=\left\lceil r\right\rceil +1,\;k=0,1,\ldots ,n-1. \end{array}\right.$$

*Then, the discrete integral equation which is equivalent to both equations in (8) is:*

(9) $$u\left(t\right)=u_{0}\left(t\right)+\frac{1}{\Gamma (r)}\sum_{s=a+n-r}^{t-r}{\left(t-s-1\right)}^{\left(r-1\right)}f\left(s+r-1,u\left(s+r-1\right)\right),$$

*where $t\in N_{a+n}$, and*

(10) $$u_{0}\left(t\right)=\sum_{k=0}^{n-1}\frac{{\left(t-a\right)}^{\left(k\right)}}{\Gamma \left(k+1\right)}\Delta^{k}u\left(a\right).$$

Using Theorem 1 and setting a=0, we get

(11) $$\left\{\begin{array}{c} x\left(n\right)=x\left(0\right)+\sum_{j=1}^{n}\frac{\Gamma (n-j+r)}{\Gamma (n-j+1)}\left(-x(j-1)+y(j-1)\right),\\ y\left(n\right)=y\left(0\right)+\sum_{j=1}^{n}\frac{\Gamma (n-j+r)}{\Gamma (n-j+1)}\left(-y(j-1)+z(j-1)\right),\\ z\left(n\right)=z\left(0\right)+\sum_{j=1}^{n}\frac{\Gamma (n-j+r)}{\Gamma (n-j+1)}(\alpha x\left(j-1\right)+\beta z\left(j-1\right)-x\left(j-1\right)y\left(j-1\right)\\ \;\;\;\;\;\;\;\;\;\;\;\;\;\;\;\;\;\;\;\;\;\;\;\;\;\;\;\;\;\;\;\;\;\;\;\;\;\;\;\;\;\;\;\;\;\;\;\;\;\;\;\;\;\;\;\;+x(j-1)z(j-1)+\gamma ), \end{array}\right.$$

where x(0),y(0) and z(0) are the initial conditions. In the next section, some numerical analyzes and simulations are presented. In particular, the effect of the fractional-order, *r*, on the behavior of the FoDT system (i.e., system (5)) will be numerically explored and discussed based on the obtained results.

## 4. Bifurcation and Coexisting Attractors

In order to characterize the dynamics of system (5), some numerical simulations related to the largest Lyapunov exponent (LE) and bifurcation diagrams are described in the next two subsections.

### 4.1. Bifurcation and Largest Lyapunov Exponent (LE)

**Figure 1:**
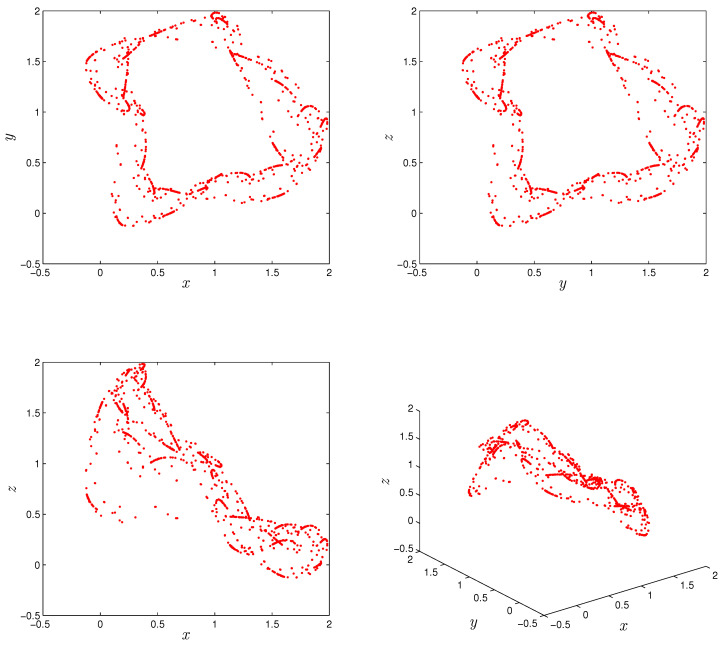
Hidden attractor of the fractional-order discrete system (5) for order r=0.999, system parameters α=1.07, β=−1.07 and γ=0.028, and initial conditions $x_{0}=0.91,y_{0}=1.63$ and $z_{0}=2.02.$

**Figure 2:**
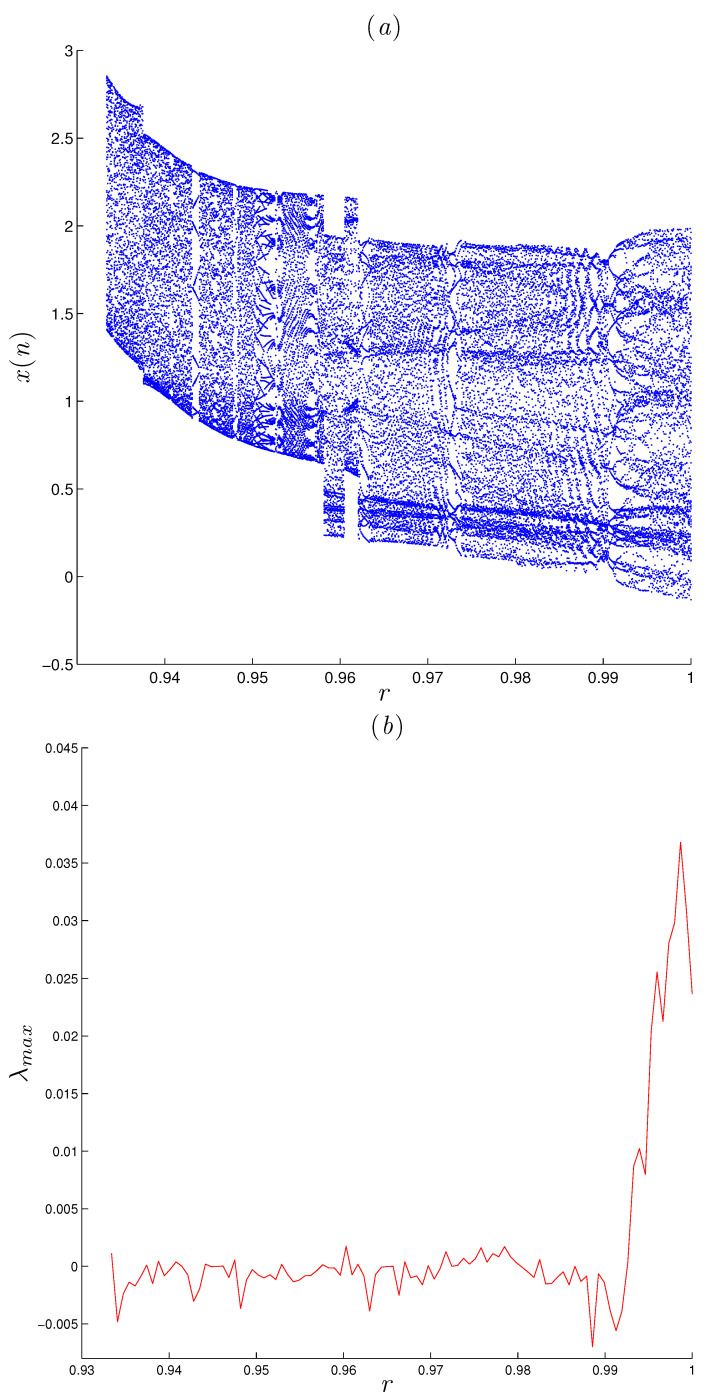
Bifurcation and the largest LE diagrams of the FoDT system (5) versus *r*, for system parameters α=1.07, β=−1.07 and γ=0.028, and initial conditions $x_{0}=0.91,y_{0}=1.63$ and $z_{0}=2.02.$ (**a**) Bifurcation diagram; (**b**) largest LE.

**Figure 3:**
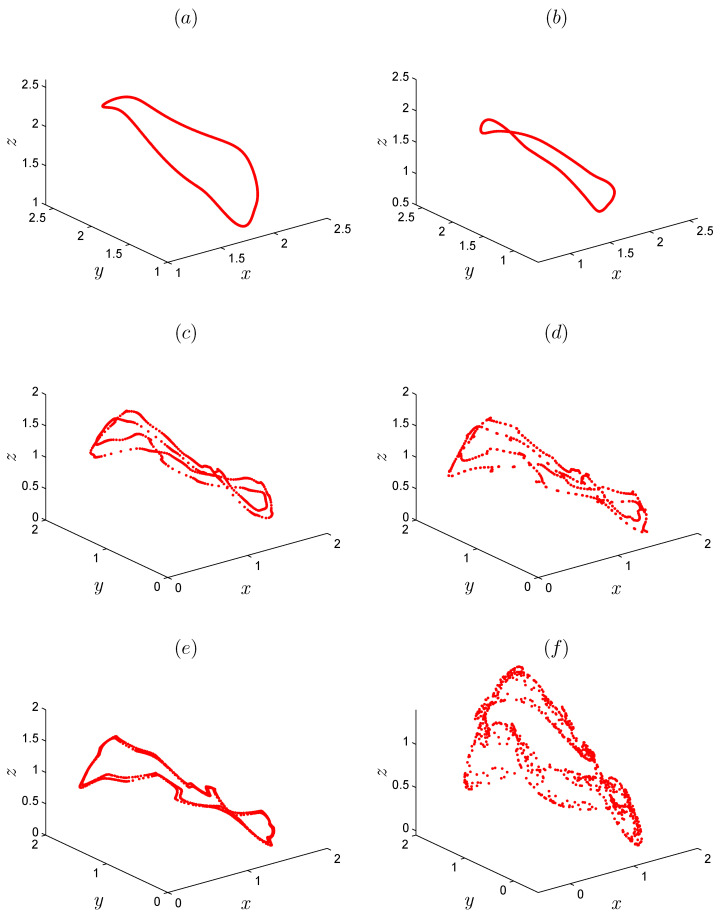
Different attractors for different fractional-order values of *r*, (**a**) r=0.9385, (**b**) r=0.95, (**c**) r=0.9633, (**d**) r=0.9868, (**e**) r=0.99, (**f**) r=0.9987.

We consider the parameter values α=1.07, β=−1.07 and γ=0.28; initial conditions $\left[x_{0},\;y_{0},\;z_{0}\right]=\left[0.91,\;1.63,\;2.02\right]$; and fractional order r=0.999. Obviously, Equation (7) has no real solution. In other words, there are no equilibrium points for the fractional system (5). Figure 1 display the hidden attractor in different phase space projections. To investigate the dynamic of the FoDT system given in (5) with respect to the fractional-order *r*, fix the system parameters as α=1.07,β=−1.07,γ=0.28 and let *r* vary from 0.93 to 1. The variation of local maxima of x(n) in the term of *r* is shown in Figure 2a. Starting from $\left[x_{0},\;y_{0},\;z_{0}\right]=\left[0.91,\;1.63,\;2.02\right]$, the states of system (5) show irregular motions along with periodic windows. We note that when r<0.9334 the FoDT system (5) diverges to infinity. To observe the dynamic behavior, the phase diagrams of the FoDT system on the 3D plane with the same initial values and for different fractional order values are shown in Figure 3. The phase diagram have been plotted so as to visualize the route to chaos. As can be seen, the FoDT system (5) has different type of hidden attractors and it goes from stable state to chaos as *r* increases.

Although bifurcation plot and phase portraits are useful tools in determining the existence of chaos and quantifying it, the most agreed upon tool is the “Lyapunov exponent.” By definition, the three-dimensional fractional-order system (5) displays sensitive dependence on initial conditions when two trajectories that are starting from infinitesimally close initial conditions can diverge exponentially with the rate given by LLE. The Lyapunov exponents can be approximated using the Jacobian matrix, which in the case of fractional-order discrete-time systems is calculated in a similar manner to the states in (11); see [31]. The Lyapunov exponents can be given by

(12) $$LE_{k}\left(x\left(0\right)\right)=\lim_{i\infty }\frac{1}{i}\ln\left|\lambda_{k}^{\left(i\right)}\right|\;\mathrm{for}\;i=\bar{1.3}$$

where $\lambda_{k}$ are the eigenvalues of the matrix $J_{i}$ defined as

(13) $$J_{i}=\left(\begin{array}{ccc} a_{i} & b_{i} & c_{i}\\ d_{i} & e_{i} & f_{i}\\ g_{i} & h_{i} & m_{i} \end{array}\right),$$

where

$$\begin{array}{cc} a_{i}= & a_{0}+\frac{1}{\Gamma (r)}\sum_{j=1}^{i}\frac{\Gamma (i-j+r)}{\Gamma (i-j+1)}\left(-a_{i}+d_{i}\right),\\ b_{i}= & b_{0}+\frac{1}{\Gamma (r)}\sum_{j=1}^{i}\frac{\Gamma (i-j+r)}{\Gamma (i-j+1)}\left(-b_{i}+e_{i}\right),\\ c_{i}= & c_{0}+\frac{1}{\Gamma (r)}\sum_{j=1}^{i}\frac{\Gamma (i-j+r)}{\Gamma (i-j+1)}\left(-c_{i}+f_{i}\right),\\ d_{i}= & d_{0}+\frac{1}{\Gamma (r)}\sum_{j=1}^{i}\frac{\Gamma (i-j+r)}{\Gamma (i-j+1)}\left(-d_{i}+g_{i}\right),\\ e_{i}= & e_{0}+\frac{1}{\Gamma (r)}\sum_{j=1}^{i}\frac{\Gamma (i-j+r)}{\Gamma (i-j+1)}\left(-e_{i}+h_{i}\right),\\ f_{i}= & f_{0}+\frac{1}{\Gamma (r)}\sum_{j=1}^{i}\frac{\Gamma (i-j+r)}{\Gamma (i-j+1)}\left(-f_{i}+m_{i}\right),\\ g_{i}= & g_{0}+\frac{1}{\Gamma (r)}\sum_{j=1}^{i}\frac{\Gamma (i-j+r)}{\Gamma (i-j+1)}\left(\left(\alpha -y\left(i\right)+z\left(i\right)\right)a_{i}-x\left(i\right)d_{i}+\left(x+\beta \right)g_{i}\right),\\ h_{i}= & h_{0}+\frac{1}{\Gamma (r)}\sum_{j=1}^{i}\frac{\Gamma (i-j+r)}{\Gamma (i-j+1)}\left(\left(\alpha -y\left(i\right)+z\left(i\right)\right)b_{i}-x\left(i\right)e_{i}+\left(x+\beta \right)h_{i}\right),\\ m_{i}= & m_{0}+\frac{1}{\Gamma (r)}\sum_{j=1}^{i}\frac{\Gamma (i-j+r)}{\Gamma (i-j+1)}\left(\left(\alpha -y\left(i\right)+z\left(i\right)\right)c_{i}-x\left(i\right)f_{i}+\left(x+\beta \right)m_{i}\right). \end{array}$$

**Figure 4:**
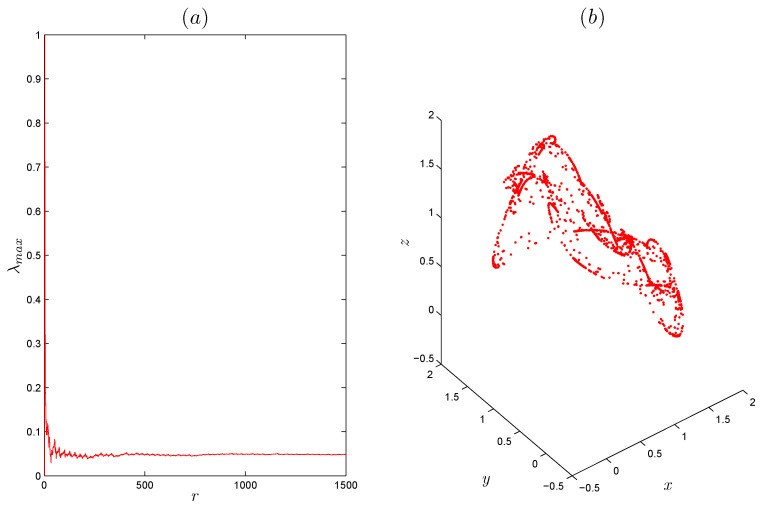
(**a**) Estimated latgest Lyapunov exponent of the fractional-order discrete system (5) for order r=0.999, α=1.07, β=−1.07 and γ=0.028, and initial conditions $x_{0}=0.91,y_{0}=1.63$ and $z_{0}=2.02.$ (**b**) Corresponding hidden chaotic attractor.

**Figure 5:**
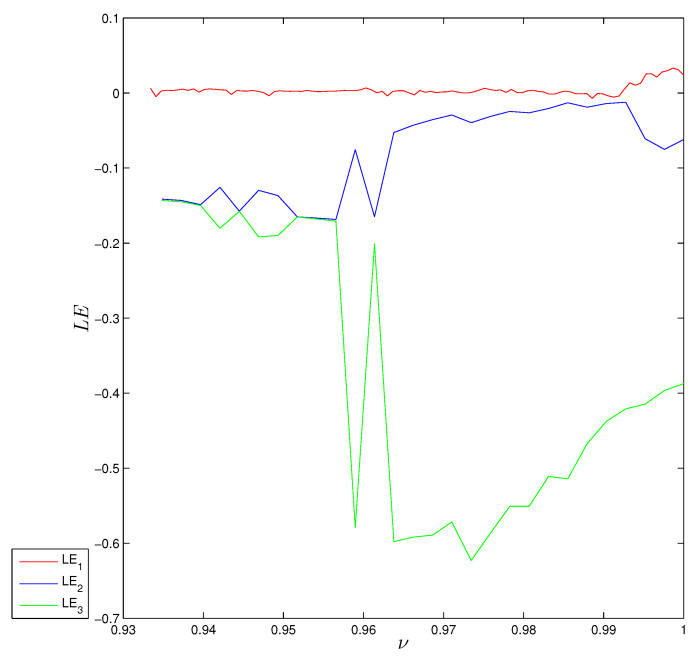
Lyapunov exponent diagrams of the FoDT system (5) versus *r*, for system parameters α=1.07, β=−1.07 and γ=0.028, and initial conditions $x_{0}=0.91,y_{0}=1.63$ and $z_{0}=2.02.$

Figure 2b shows the largest LE of system (5) that was calculated using the above method with MATLAB, from which it can be seen that the system has positive LEs when *r* takes its highest values, indicating that the system converges to hidden chaotic attractors, which is consistent with the corresponding bifurcation diagram. LEs are negative or equal to zero for the remaining ranges, which indicates that the system has limited cycles and periodic attractors, as shown in Figure 3. Taking r=0.99 as an example, Figure 4a shows the estimated largest Lyapunov exponent and the phase portrait, respectively. Obviously, the corresponding largest Lyapunov exponent is positive, which shows the the fractional system (5) is chaotic in this case. All Lyapunov exponents are plotted in Figure 5 for varied *r* to validate these results.

**Figure 6:**
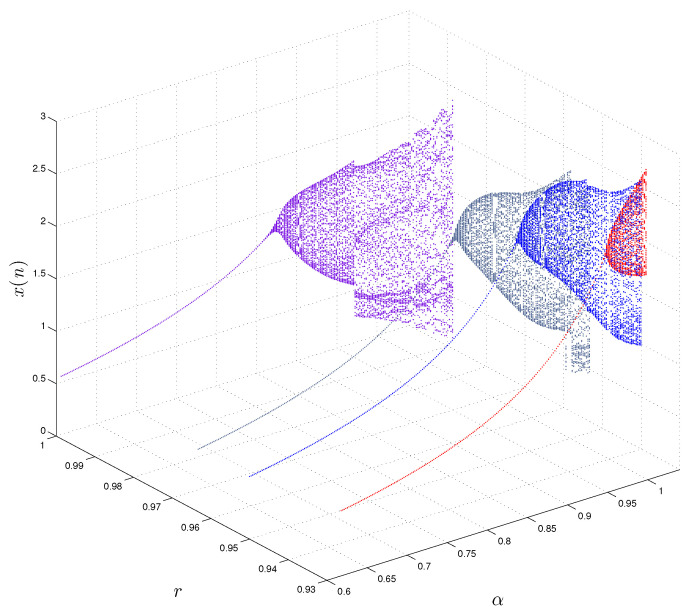
Different bifurcation diagrams of the FoDT system (5) in three-dimensional space with the variation of system parameter α and fractional order *r*.

**Figure 7:**
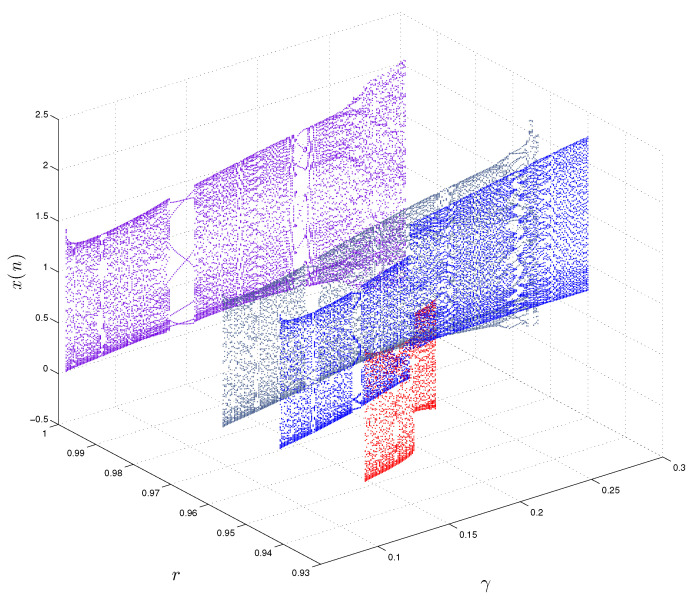
Different bifurcation diagrams of the FoDT system (5) in three-dimensional space with the variation of system parameter γ and fractional order *r*.

In order to further analyze the properties of the FoDT system (5), we plot the bifurcation diagrams for different values of *r* (i.e., r=0.93,r=0.95,r=0.9633,r=0.9987). In Figure 6 and Figure 7 we show the bifurcation diagram of the FoDT system (5) with respect to the α and γ, respectively. In Figure 6, the diagrams are obtained by changing α from 0.6 to 1.088 and fixing β and γ to β=−1.07,γ=0.28, while in Figure 7 we present the 3D view of the bifurcation diagrams when γ∈(0.05,0.3). Different dynamic behavior, including chaos periodic windows, is observed. Moreover, we observe that decreasing the value of the fractional order lead to the disappearance of some chaotic region. In particular, the diagrams of bifurcation in Figure 6 and Figure 7 show that the bifurcation diagram of the FoDT system (5) shrinks as *r* decreases.

### 4.2. Coexisting Attractors

**Figure 8:**
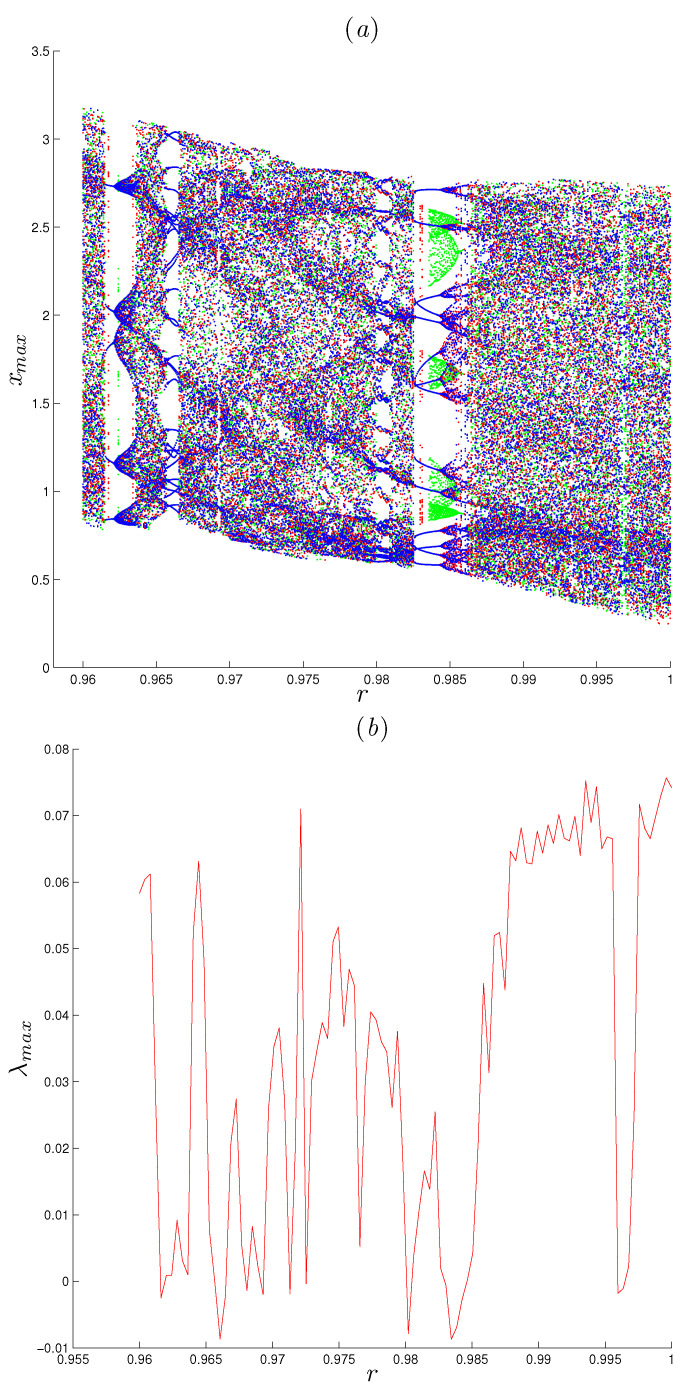
Bifurcation diagram of system (5) versus *r* depicted in (**a**), for parameters α=1, β=−1 and γ=0.5 and initial conditions ((0.97,0.54,0.23) for the blue diagram and (−0.97,0.54,0.23) for the green diagram and (0.97,0.54,−0.23)) for the red diagram. (**b**) The corresponding largest Lyapunov exponent of system (5) for initial conditions (0.97,0.54,0.23).

**Figure 9:**
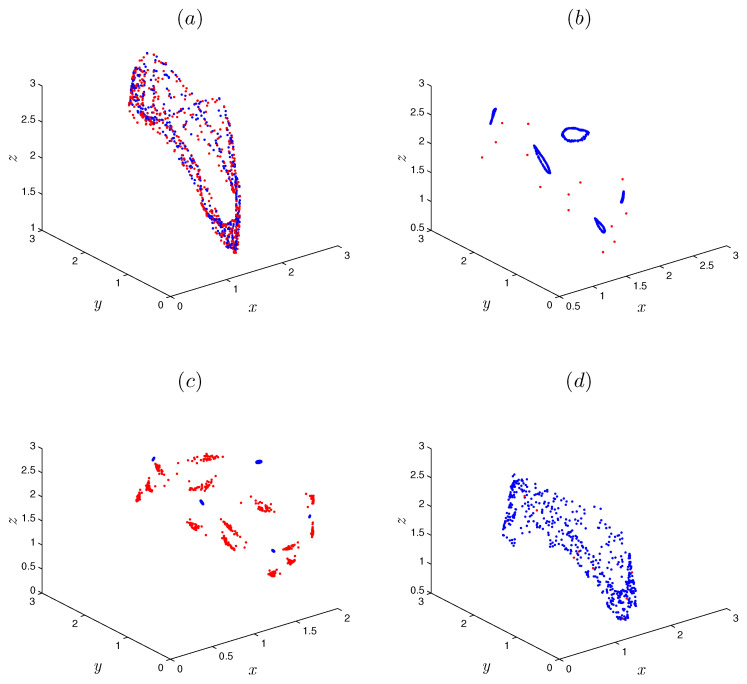
The coexisting attractors of FoDT system (5) with parameters α=1, β=−1 and γ=0.5, and with the initial conditions (0.97,0.54,0.23) for the red attractor and (−0.97,0.54,0.23) for the blue attractor. (**a**) Two coexisting periodic attractors for r=0.9615. (**b**) Periodic orbit and 5 limit cycles for r=0.984. (**c**) Two coexisting periodic attractors for r=0.9855. (**d**) Chaotic attractor and periodic orbit for r=0.996.

Here, the dynamics of the FoDT system given in (5) are analyzed using the coefficient α=1,β=−1 and γ=0.5, with three different values of initial conditions. The coexisting bifurcation diagram, shown in Figure 8a, is obtained by varying *r* in the interval [0.96,1], where the blue, green and red diagrams are drawn according to the initial conditions [0.97,0.54,0.23], [−0.97,0.54,0.23] and [0.97,0.54,−0.23], respectively. Regarding with *r* again, the behavior of dynamical system (5) can be exhibited by the largest LE graph shown in Figure 8b. One can observe that when r∈[0.96,1], the largest LE changes its values between positive and negative numbers, which indicates that there is a transition from chaos to periodic to chaos with the various values of *r*. When r∈[0.9869,1], system (5) is chaotic as its largest LE is positive. At r=0.9969, system (5) exhibits periodic motions. Moreover, when we choose r=0.996, we can obtain a chaotic attractor for the initial condition [0.97,0.54,0.23] and a periodic orbit for [−0.97,0.54,0.23], as shown in Figure 9d. Further, as *r* decreases through the interval [0.9826,0.9863], system (5) has two different types of attractors coexist with respect to the initial condition [0.97,0.54,0.23] and [−0.97,0.54,0.23], as shown in Figure 9c.

## 5. 0–1 Test and Approximate Entropy (AnEn)

In this section, the chaotic behavior of system (5) is tested using two chaotic tests: the 0–1 test and the ApEn test. Such tests are added to quantify the amount of regularity and the unpredictability in the FoDT system over the state data.

### 5.1. 0–1 Test

To detect the existence of chaos for deterministic systems, the 0–1 test is considered one of the newest suitable tools that can be employed. It is applied on a series of data that can be originated from such systems, such as the FoDT one. For system (5), consider a set of data x(j), where j=1,2,…,N. By following [32], one can define the two terms; $p_{c}\left(n\right)=\sum_{j=1}^{n}x\left(j\right)\cos\left(jc\right)$ and $q_{c}\left(n\right)=\sum_{j=1}^{n}x\left(j\right)\sin\left(jc\right)$, for n=1,2,…,N. Such terms are called the translation components, where *c* could be randomly chosen from the interval (0,2π). To determine whether chaos occurs, we can simply plot $q_{c}$ and $p_{c}$ in the two-dimensional $\left(p_{c}-q_{c}\right)$ plane. Namely, the Brownian-like trajectories imply chaos, whereas the bounded-like trajectories imply regular dynamics. The mean square displacement, which can be defined as $M_{n}=\frac{1}{N}\sum_{j=1}^{N}\left({\left(p(j+n)-p(j)\right)}^{2}+{\left(q(j+n)-q(j)\right)}^{2}\right)$, might be calculated in order to investigate the diffusive behavior of p(n) and q(n). Finally, we obtain the asymptotic growth rate *K* via $K_{c}=median\left(K_{c}\right)$, where $K_{c}=\lim_{n\to +\infty }\frac{\log M_{c}}{\log n}.$ Therefore, the proposed system becomes chaotic as *K* approaches 1, while it becomes periodic as *K* approaches 0. Table 1, however, depicts the results of the test for different values of *r*, in which $\left[x_{0},\;y_{0},\;z_{0}\right]=\left[0.97,\;0.54,\;0.23\right]$ and α=1,β=−1,γ=0.5. Based on said table, one can observe that the output *K* has appeared in a similar manner to the results of the largest LE and bifurcation diagram, shown in Figure 8. Furthermore, as the FoDT system becomes chaotic, the growth rate, *K*, asymptotically approaches 1.

**Figure 10:**
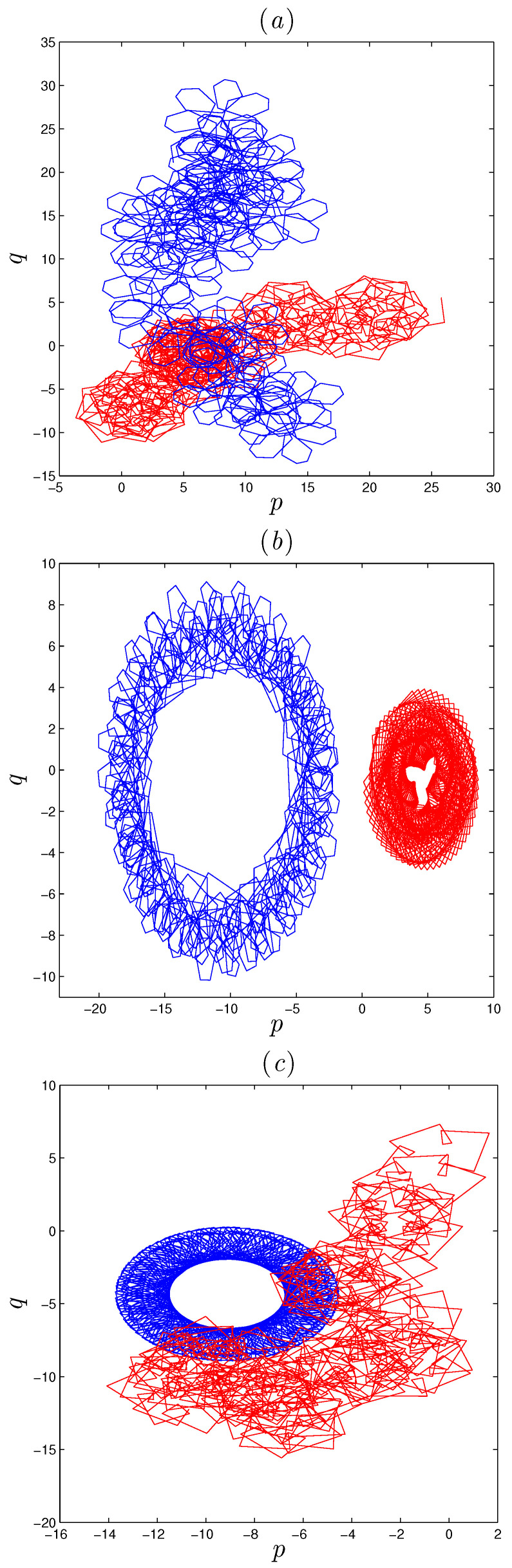
0–1 test of the FoDT system (5): (**a**) Brownian-like trajectories for r=0.9855, (**b**) bounded-like trajectories for r=0.9935, (**c**) Brownian-like trajectories for the initial conditions (0.97,0.54,0.23) and r=0.996; bounded–like trajectories for the initial conditions (−0.97,0.54,0.23) and r=0.996.

The coexistence of chaotic attractors in system (5) can be validated by plotting the translation components of this test in a plane in which *p* and *q* are its axises. As in Figure 9, we have fixed the system’s parameters α,β and γ equal to 1,−1 and 0.5 respectively, and have constantly varied *r*. Figure 10 illustrates the results of the 0–1 test for system (5) in which the blue and the red diagrams are drawn according to the two initial conditions $\left[x_{0},\;y_{0},\;z_{0}\right]=\left[-0.97,\;0.54,\;0.23\right]$ and $\left[x_{0},\;y_{0},\;z_{0}\right]=\left[0.97,\;0.54,\;0.23\right]$, respectively. In particular, Figure 10a depicts Brownian-like trajectories for both initial conditions, confirming the coexistence of two chaotic attractors for r=0.9615. On the other hand, Figure 10b depicts bounded-like trajectories for the same two initial condition, confirming the coexistence of two periodic attractors for r=0.984. Finally, the coexistence of chaotic and periodic orbits is confirmed in Figure 10c, which depicts bounded-like trajectories for initial condition [−0.97,0.54,0.23] and Brownian-like trajectories for [0.97,0.54,0.23], when r=0.996.

### 5.2. Approximate Entropy (ApEn)

The so-called ApEn was first revealed by Pincus in [33] to measure the degree of complexity of data in a time series from a multi-dimensional perspective. This method of testing is used to estimate the regularity of some data and assign a non-negative number to a certain sequence, in which, e.g., these data become more complex when the values of them being large. The detailed calculation steps for detecting ApEn are stated as follows. Consider x(1),x(2),…,x(n) as a set of *n* discrete data deduced based on the FoDT system given in (5). The value of the ApEn depends on two important parameters, *m* and τ, where the input τ is the similar tolerance and *m* is the embedding dimension. For a given *m*, form a sequence of vectors X(j), for j=1,2,…,n−m+1, as X(j)=(x(j),…,x(j+m−1)). Let *K* the number of X(i) be such that the maximum absolute difference of two vectors, X(i) and X(j), is equal or lower than τ. The relative frequency of X(i) is similar to X(j), and it has the form: $C_{i}^{m}\left(\tau \right)=\frac{K}{N-m+1}.$ From $C_{i}^{m}$, calculate the logarithm and then define the average for all *i*, as follows:

(14) $$\phi^{m}\left(\tau \right)=\frac{1}{N-m-1}\sum_{i=1}^{N-m+1}\log C_{i}^{m}\left(\tau \right).$$

The ApEn of order *m* is, then, as follows:

(15) $$ApEn=\phi^{m}\left(\tau \right)-\phi^{m+1}\left(\tau \right).$$

**Figure 11:**
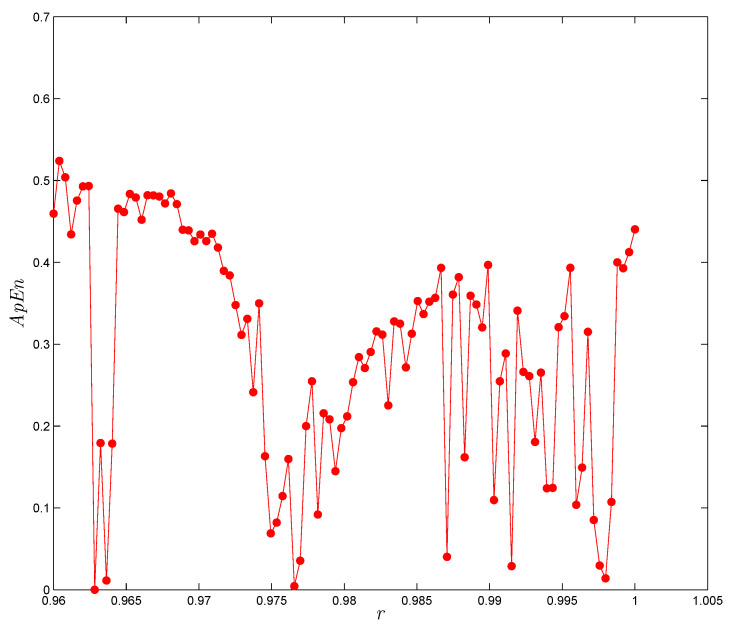
ApEn of the FoDT system (4) versus *r*, for system parameters α=1, β=0 and γ=0.5, and initial conditions (0.97,0.54,0.23).

Table 2 and Figure 11 show the ApEn of the proposed FoDT system given in (5) for various values of *r*, when α=1, β=−1 and γ=0.5, and according to $x_{0}=0.97$, $y_{0}=0.54$ and $z_{0}=0.23$. As one can see, the complexity of this system varied according to the variation of *r*. Hence, one should be conscious in selecting the values of *r* in system (5), in order to obtain a relatively large structural complexity.

## 6. One-Dimensional Control Law

Here, we need to stabilize all states of the FoDT system and eliminate the chaotic motion by adding an adaptive controller. In fact, this controller will regulate the trajectories generated by system (5) to the zero equilibrium point. However, the following theorem is stated to discuss the stability analysis of such system.

**Theorem** **2.** *The equilibrium at zero of the linear FoDT system:*

(16) $${}^{C}\Delta_{a}^{r}X\left(t\right)=MX\left(t+r-1\right),$$

*is asymptotically stable if*

(17) $$\lambda \in \left\{z\in C:\;\left|z\right|<{\left(2\cos\frac{\left|\arg z\right|-\pi }{2-r}\right)}^{r}\mathrm{and}\;\left|\arg z\right|>\frac{r\pi }{2}\right\},\forall \lambda$$

*where $X\left(t\right)={\left(x_{1}\left(t\right),\ldots ,x_{n}\left(t\right)\right)}^{T}$; $\forall t\in N_{a+1-r}$, 0<r≤1, $M\in R^{n\times n}$ and where λ is the eigenvalue of M.*

For the proof of this theorem in detail, refer to [34]. However, the controlled three-dimensional FoDT system given in (5) will be:

(18) $$\left\{\begin{array}{c} {}^{C}\Delta_{a}^{r}x\left(t\right)=-x\left(t-1+r\right)+y\left(t-1+r\right),\\ {}^{C}\Delta_{a}^{r}y\left(t\right)=-y\left(t-1+r\right)+z\left(t-1+r\right),\\ {}^{C}\Delta_{a}^{r}z\left(t\right)=\alpha x\left(t-1+r\right)+\beta z\left(t-1+r\right)-x\left(t-1+r\right)\\ \;\;\;\;\;\;\;\;\;\;\;\;\;\;\;\;\;\;\;\;\;\;\;\;\;\;\;\;\;\;\;\;\;\;\;\;\;\;\;\;\;\;\;\;\;\;\;\;\;\;\;\;\;\left[z(t-1+r)-y(t-1+r)\right]+\gamma +C\left(t\right), \end{array}\right.$$

where C(t) denotes the one-dimensional controller.

Our goal here is to find a suitable one-dimensional controller that can stabilize all states of the FoDT system towards zero asymptotically. For that purpose, we propose the following theorem.

**Theorem** **3.** *The following control law of one dimension:*

(19) $$C\left(t\right)=-\alpha x\left(t\right)+0.07z\left(t\right)+x\left(t\right)\left[z(t)-y(t)\right]-\gamma ,$$

*can stabilize the three-dimensional FoDT system given in (5).*

**Proof.**  Substituting (19) in the third state of the FoDT system given in (18) yields:

(20) $$\begin{array}{c} {}^{C}\Delta_{a}^{r}{\left(x\left(t\right),y\left(t\right),z\left(t\right)\right)}^{T}=\\ \;\;\;\;\;\;\;\;\;\;\;\;\;\;\;\;\;\;\;\;\;\;\;\;\;\;\;\;\;\;\;\;\;\;\;\;\;\;\;M\times \left(x\left(t-1+r\right),y\left(t-1+r\right),z(t-1+r\right){)}^{T}, \end{array}$$

where

(21) $$M=\left(\begin{array}{ccc} -1 & 1 & 0\\ 0 & -1 & 1\\ 0 & 0 & \beta +0.7 \end{array}\right).$$

Now, one needs to show that all states of the controlled system converge to zero and stay there. The eigenvalues $\lambda_{1},$$\lambda_{2}$ and $\lambda_{3}$ of the matrix M have been found; they satisfy the mentioned condition in Theorem 2. That is,

$$\left|\arg\lambda_{i}\right|=\pi >\frac{r\pi }{2}\;\mathrm{and}\;\left|\lambda_{i}\right|=1<{\left(2\cos\frac{\left|\arg\lambda_{i}\right|-\pi }{2-r}\right)}^{r},\;i=1,2,3.$$

Hence, the zero equilibrium of (20) is asymptotically stable, which implies that the proposed three-dimensional discrete-time system given in (5) has been stabilized. □

**Figure 12:**
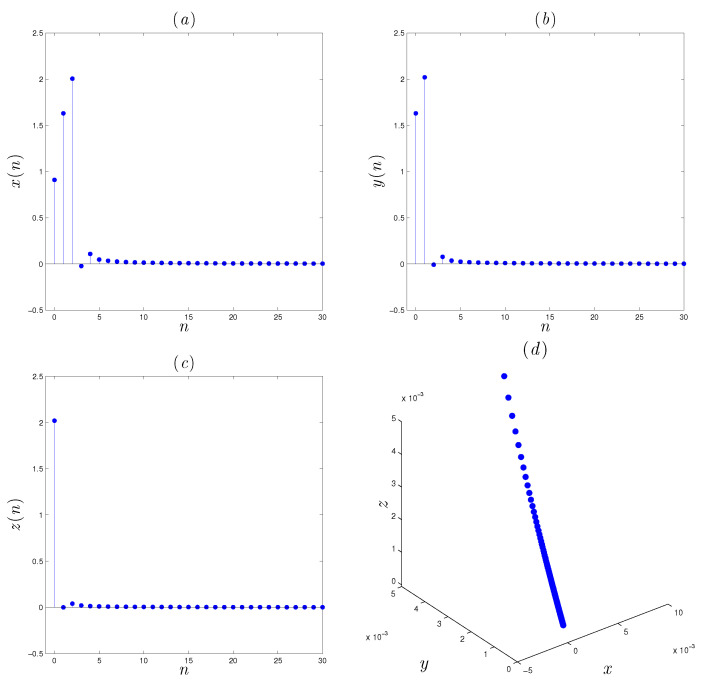
Evolution of the controlled fractional-order system (5) and phase portrait: (**a**) evolution of the first state x(n), (**b**) evolution of state y(n), (**c**) evolution of state z(n), (**d**) phase portrait.

To confirm the above theoretical results, some numerical simulations of the controlled system are exhibited in Figure 12. The parameters of system (5), in this figure, are taken to be as $\left(\alpha ,\;\beta ,\;\gamma \right)=\left(1.07,\;-1.07,\;0.28\right)$, and *r* is chosen to be equal 0.98. Based on such simulation, we can easily observe that the controller has compelled the states towards zeros as required.

## 7. Conclusions

A new three-dimensional, fractional-order discrete-time (FoDT) system has been examined in this article based on the Caputo-type delta difference operator. Through phase portraits, bifurcation diagrams and largest LE, the existence of the chaos for such a fractional map has been explored, as has the type of the chaotic behavior and its range. It has been verified that this chaotic behavior and its range depend on the values of fractional order. Further, all numerical examinations have demonstrated that the system exhibits the property of coexisting attractors. In this work, the 0–1 test succeeded in testing the chaotic behavior of the system, and an approximate entropy (ApEn) tool has been utilized to measure the complexity of the system. In order to stabilize the FoDT system, a valid control method of stabilization has been also proposed in such a way as to force all its states to be adaptively tended toward an equilibrium point at zero. The linear stability theory of the FoDT systems has been utilized to conclude that the equilibrium of the proposed controlled map is asymptotically stable at a zero solution. For the duration of this work, all findings and results have been confirmed and explained by implementing suitable numerical simulations. We will focus on putting this claim to the test in a future study. An interesting goal is that of developing a new type of fractional pseudonumber generator based on the fractional maps with hidden attractors for potential cryptographic application, and another is implementing the previous model in terms of modeling electrical circuits. It is our intention to investigate this issue further in future studies.

## Figures and Tables

**Table 1:** 0–1 test of the FoDT system (5) for different values of r.

*r*	0.9604	0.9615	0.9632	0.9739	0.984	0.996
K	0.957	0.8178	0.0052	0.9356	0.0027	0.9934

**Table 2:** Approximate entropy (ApEn) of the FoDT system (5) for different values of r.

*r*	0.9604	0.9615	0.9632	0.9739	0.984	0.996
ApE	0.3928	0.2037	0.1494	0.3933	0.069	0.4928
